# A comparative study of dipolarization fronts at MMS and Cluster

**DOI:** 10.1002/2016GL069520

**Published:** 2016-06-25

**Authors:** D. Schmid, R. Nakamura, M. Volwerk, F. Plaschke, Y. Narita, W. Baumjohann, W. Magnes, D. Fischer, H. U. Eichelberger, R. B. Torbert, C. T. Russell, R. J. Strangeway, H. K. Leinweber, G. Le, K. R. Bromund, B. J. Anderson, J. A. Slavin, E. L. Kepko

**Affiliations:** ^1^ Space Research InstituteAustrian Academy of SciencesGrazAustria; ^2^ NAWI GrazUniversity of GrazGrazAustria; ^3^ Institute for the Study of Earth, Oceans, and SpaceUniversity of New HampshireDurhamNew HampshireUSA; ^4^ Southwest Research InstituteSan AntonioTexasUSA; ^5^ Institute of Geophysics and Planetary PhysicsUniversity of CaliforniaLos AngelesCaliforniaUSA; ^6^ NASA Goddard Space Flight CenterGreenbeltMarylandUSA; ^7^ The Johns Hopkins Applied Physics LaboratoryLaurelMarylandUSA; ^8^ Department of Climate and Space Sciences and EngineeringUniversity of MichiganAnn ArborMichiganUSA

**Keywords:** magnetotail, dipolarization front, MMS, Cluster

## Abstract

We present a statistical study of dipolarization fronts (DFs), using magnetic field data from MMS and Cluster, at radial distances below 12 *R*_*E*_ and 20 *R*_*E*_, respectively. Assuming that the DFs have a semicircular cross section and are propelled by the magnetic tension force, we used multispacecraft observations to determine the DF velocities. About three quarters of the DFs propagate earthward and about one quarter tailward. Generally, MMS is in a more dipolar magnetic field region and observes larger‐amplitude DFs than Cluster. The major findings obtained in this study are as follows: (1) At MMS ∼57 % of the DFs move faster than 150 km/s, while at Cluster only ∼35 %, indicating a variable flux transport rate inside the flow‐braking region. (2) Larger DF velocities correspond to higher *B*_*z*_ values directly ahead of the DFs. We interpret this as a snow plow‐like phenomenon, resulting from a higher magnetic flux pileup ahead of DFs with higher velocities.

## Key Points

- MMS is generally located in a more dipolar magnetic field region and observes larger‐amplitude DFs than Cluster farther down the tail
- A larger fraction of DFs move faster closer to Earth, suggesting variable flux transport rates in the flow‐braking region
- Larger DF velocities correspond to a higher Bz directly ahead of DFs, suggesting a higher flux pileup ahead of DFs with higher velocities

## 1 Introduction

The Earth's magnetotail consists of two lobe regions of stretched, oppositely directed magnetic fields separated by a high‐*β* plasma/current sheet with an embedded neutral sheet. When oppositely directed magnetic field lines reconnect in the magnetotail, the relaxation of the magnetic tension of the stretched field lines converts the stored magnetic energy into plasma kinetic energy and heat. The magnetoplasma is accelerated earthward in short duration bursty bulk flows (BBFs) [*Angelopoulos et al.*, 1992; *Baumjohann et al.*, 2002]. The BBFs are the most prominent means to carry mass and energy from the tail toward the near‐Earth region. BBFs are often accompanied by magnetic field dipolarizations [e.g., *Nakamura et al.*, 2002, 2009]. Observationally, they are seen by satellites as a sharp increase in the vertical‐to‐the‐current sheet component (*B*_*z*_), usually preceded by a transient decrease in *B*_*z*_ [e.g., *Ohtani et al.*, 2004]. These asymmetric bipolar variations in the *z* component of the magnetic field are referred to as dipolarization fronts (DFs) [*Nakamura et al.*, 2002; *Runov et al.*, 2011; *Schmid et al.*, 2011; *Fu et al.*, 2012a].

DFs are also interpreted as thin boundary layers of earthward moving flux tubes, which have a reduced entropy compared to the ambient plasma in the tail [e.g., *Pontius and Wolf*, 1990]. As long as the entropy of the flux tube is lower, it can continue to propagate earthward, and it stops when both are equal [e.g., *Sergeev et al.*, 2012]. The pressure balance of these structures with the ambient plasma is maintained by the stronger magnetic field within the flux tube [see, e.g., *Li et al.*, 2011]. According to *Liu et al.* [2013] we call this stronger magnetic region, led by the DF, as dipolarizing flux bundle (DFB). DFs have a typical thickness, which is on the order of the ion inertial length [e.g., *Runov et al.*, 2011; *Schmid et al.*, 2011; *Fu et al.*, 2012b; *Huang et al.*, 2012], and they move as coherent structures over macroscopic distances (several hundred ion inertial lengths) [*Runov et al.*, 2009]. However, a simplified picture of a gradually stopping flux tube does not always match observations. *Panov et al.* [2010] showed a change in the flow burst propagation direction that suggests a rebound (bouncing) of the DF at the magnetic dipole‐dominated near‐Earth plasma sheet. It was predicted by *Chen and Wolf* [1999] that the earthward moving DFs can overshoot their equilibrium position, after which they will perform a damped oscillation. Indeed, simulations [e.g., *Birn et al.*, 2011] and observations [e.g., *Schmid et al.*, 2011; *Zhou et al.*, 2011; *Nakamura et al.*, 2013; *Huang et al.*, 2015] show that DFs propagate not only earthward but also tailward.

In this paper, we use Magnetospheric Multiscale (MMS) mission magnetotail observations and compare and contrast the identified DFs with DF observations from the Cluster mission. With MMS at radial distances within 12*R*_*E*_ and Cluster at ∼19 *R*_*E*_, it is for the first time possible to compare the inner and outer magnetotail regions using multispacecraft observations of DFs.

## 2 Data and Event Selection

For this study, we use MMS magnetic field observations from the Earth's magnetotail, between April and July 2015. During this period the mission was still in the commissioning phase and only the flux‐gate magnetometers (FGMs) [*Russell et al.*, 2014; *Torbert et al.*, 2014] were operating continuously. For commission the digital flux‐gate magnetometers (DFGs) 128 Hz data are available almost over the entire period.

For the DF event selection the high‐resolution data are down‐sampled to 1Hz, because of the large amount of data. However, after the DF survey we use the high‐resolution data for the analysis. To find the DFs, we apply the selection criteria introduced in *Schmid et al.*[2011] without the criteria on the plasma quantities, due to the limited amount of plasma data available. Within 3 min long sliding windows shifted by 30 s; the following criteria should be fulfilled:

1. The spacecraft is located in the magnetotail between *X*_GSM_≤−5*R*_*E*_ and |*Y*_GSM_|≤15*R*_*E*_.
2. The difference in elevation angle $\left(\theta =\arctan\left(B_{z}/B_{\mathrm{xy}}\right)\right)$ between minimum and maximum *B*_*z*_ during the window exceeds 10° and *Δ* *B*_*z*_ also exceeds 4nT.
3. The arrival time of the maximum *B*_*z*_ is later than that of the minimum *B*_*z*_.
4. The elevation angle is at least in one data point (within the 3 min window) greater than $\theta_{\max}\geq 45$°.

These selection criteria are applied to each spacecraft and only events observed by all four MMS satellites are selected. An automatic routine identified 201 DF events between April and July 2015 at radial distances within 12*R*_*E*_.

We compare the MMS DF events with DF observations from Cluster in the season from July and October 2003. During that time Cluster had similar interspacecraft distances (∼200 km), but the spacecraft were located at larger radial distances (∼19 *R*_*E*_). We start from the existing Cluster DF event catalog introduced in *Schmid et al.* [2015], which is based on the same selection criteria on the magnetic field data. We up‐sample the burst mode flux‐gate magnetometer (FGM) [*Balogh et al.*, 1997] data to 128 Hz. It should be noted that the DFs in this list also satisfy criteria on the plasma data (|*V*_*x*_|≥100 km/s, spacecraft (S/C) within the plasma sheet, see Appendix A in *Schmid et al.* [2015]). Here we select only events observed by all four Cluster spacecraft within |*Z*_GSM_|≤5*R*_*E*_ during 2003. These add up to 110 DFs.

For each of the 201 MMS and 110 Cluster events, a 3 min interval is selected, which is centered on the minimum value of *B*_*z*_ (set to *t* = 0 s). At this point the sharp increase in *B*_*z*_ (dipolarization) starts. On the magnetic field between the minimum and maximum values of *B*_*z*_ a minimum variance analysis (MVA) [*Sonnerup and Scheible*, 1998] is performed, which gives the normal direction to the DF. Also, the following requirements are added to the events:

1. The ratio of the intermediate to minimum eigenvalues shall be $\lambda_{\text{int}}/\lambda_{\min}\geq 4$ to ensure a minimum confidence level while keeping the sample size large enough for our statistical study [see, e.g., *Sergeev et al.*, 2006].
2. Assuming the DF has a saddle‐like shape (semicircular geometry in *X* *Y* plane) and is stable during the DF passage over all spacecraft, the estimated normal direction to the front from each spacecraft shall differ by at most 15°, to ensure that each spacecraft crosses the DF almost at the same location.
3. To minimize the projection errors in the DF velocity determination, we require the S/C to cross the DF around its center (the angle between assumed propagation direction (see section 3) and the S/C crossing normal vector shall be smaller than 45°).
4. To accurately determine the time delay between the S/C, and thus the DF velocity, we require all S/C to observe very similar magnetic signatures by visual inspection, to ensure reliable cross‐correlation time lags.

Therewith, 23 DFs (out of 201) represent the MMS data set for our study and 23 DFs (out of 110) the Cluster data set. The list of DFs is provided in the supporting information.

**Figure 1:**
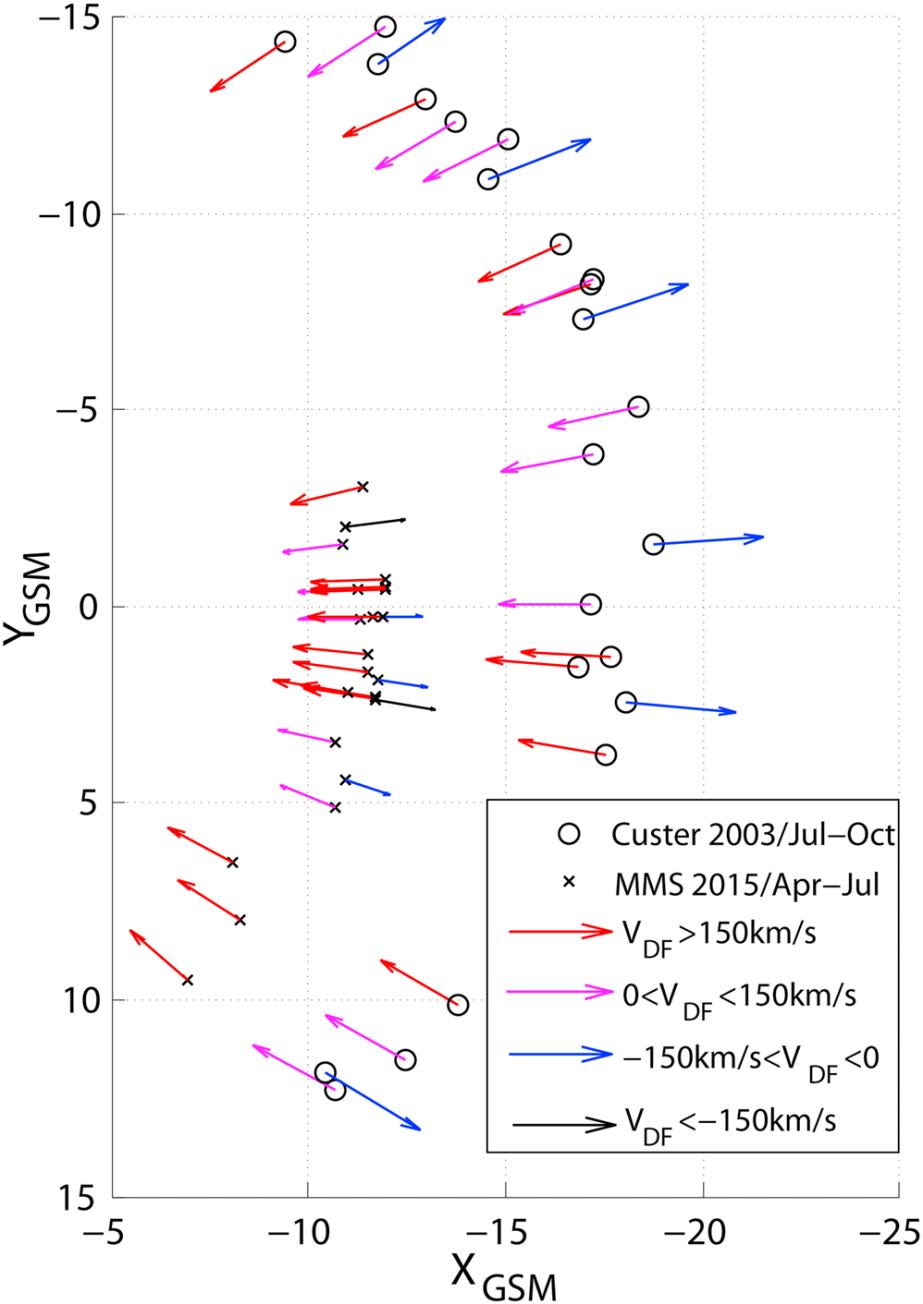
*X* *Y* position of MMS (stars) and Cluster (dots) during the observations of the DF events. The colored arrows indicate the earthward/tailward DF propagation directions and velocities as of the four velocity bins.

The distribution of the 23 MMS and 23 Cluster DFs on the *X* *Y* plane in the GSM coordinate system is shown in Figure 1. Crosses and circles in black mark the barycenter positions of MMS and Cluster, respectively. The colored arrows indicate the earthward/tailward DF propagation directions and velocities.

MMS observes more events in the premidnight sector as the commissioning orbits do not cover postmidnight equally well.

## 3 Observations and Methodology

A new coordinate system, the T89 coordinate system {*X*_T89_,*Y*_T89_,*Z*_T89_}, introduced by *Schmid et al.* [2015], is used, which is based on the magnetic field model by *Tsyganenko* [1989]. In the T89 system, *X*_T89_ is in the direction of the magnetic tension force and is determined by the average direction in the northern and southern lobe ±3*R*_*E*_ away in the *Z*_GSM_ direction from the spacecraft location projected on the *X* *Y* GSM plane and is positive toward the Earth. *Z*_T89_ points along *Z*_GSM_ and *Y*_T89_=*Z*_T89_×*X*_T89_ completes the right‐handed coordinate system.

We assume the DFs to propagate along *X*_T89_ as they should be propelled by the magnetic tension force. Hence, the DF propagation directions point radially inward or outward to/from the Earth, as can be seen in Figure 1.

**Figure 2:**
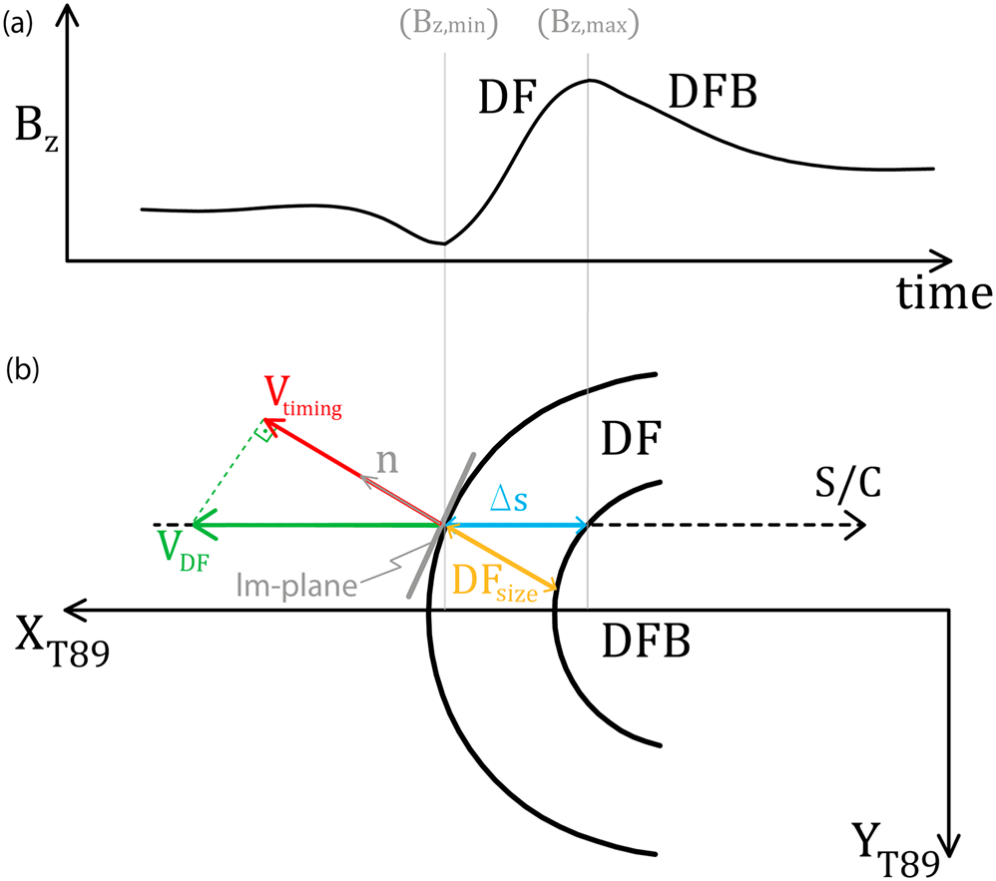
Illustration of (a) S/C in situ observations of the magnetic field *Z* component (*B*_*z*_), (b) assumed circular shape of the DF in the *X* *Y* plane. **n** denotes the normal direction where the S/C crossed the front. *V*_timing_ is the velocity of the magnetic structure, obtained by the timing method. *V*_DF_ is the DF velocity along the assumed propagation direction *X*_T89_. *Δ* *s* is the observed front thickness (between $B_{z,\min}$ and $B_{z,\max}$) and DF_size_ the actual DF thickness.

Figures 2a and 2b illustrate S/C in situ observations of *B*_*z*_ and the assumed circular shape of the DFs in the *X* *Y* plane, respectively. The point **n** denotes the normal direction where the S/C crossed the front. *V*_timing_ is the velocity along the crossing normal direction determined from the timing method: To determine the time lag between the S/C observations (and thus the normal velocity) accurately, the magnetic field *B*_*z*_ data between $B_{z,\min}$ and $B_{z,\max}$ of those two S/C which are farthest apart along **n** are cross‐correlated. On the assumption that the DFs propagate along *X*_T89_, it is possible to estimate the DF velocity (*V*_DF_ in Figure 2b). We then estimate the thickness of the DFs using their velocities and crossing durations (DF_size_ in Figure 2b).

## 4 Statistical Analysis

**Figure 3:**
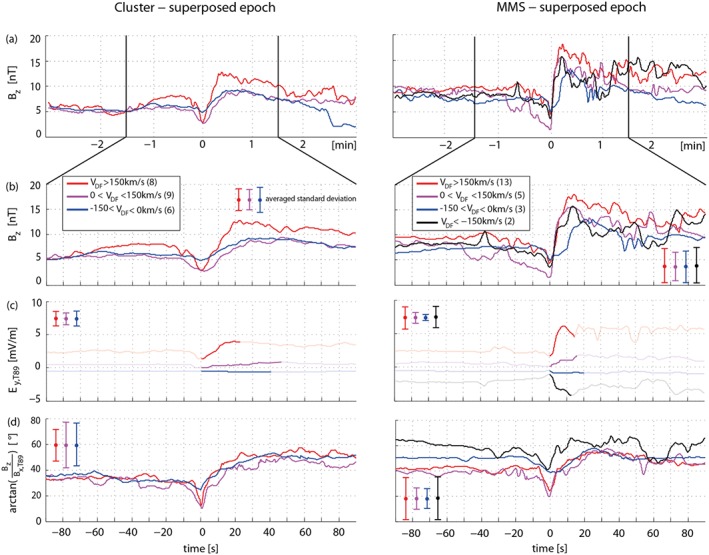
Superposed Epoch analysis of (a and b) *B*_*z*_, (c) motional electric field, and (d) the magnetic elevation angle of the DFs observed by Cluster (left) and MMS (right). The 23 Cluster and 23 MMS events are divided into four subsets according to their DF velocity. The number of events in each bin is given in the legend.

Figure 3 shows the superposed epoch analysis for the 23 Cluster (Figure 3, left) and 23 MMS (Figure 3, right) events. The data are smoothed by averaging over 128 data points (1 s of data). Figure 3a shows the *z* component of the magnetic field ±3min around the DF onset. Figures 3b–3d show the superposed epoch for *B*_*z*_, the motional electric field *E*_*y*,T89_, and the magnetic elevation angle, 90s around the DF onset, respectively. The motional electric field is obtained from *E*_*y*,T89_=*V*_DF_ *B*_*z*_. Since *E*_*y*,T89_ is obtained from the DF velocity, only the values determined between $B_{z,\min}$ and $B_{z,\max}$ are reliable (thick lines). A higher *B*_*z*_ at higher velocities leads to a higher *E*_*y*,T89_, which indicates a higher flux transport rate toward the Earth. The magnetic elevation angle is given by $\arctan\left(B_{z}/B_{x,\text{T89}}\right)$. To examine how *B*_*z*_ changes in association with the DF velocity, each data set is divided into four subsets: *V*_DF_<−150km/s (black), −150km/s < *V*_DF_<0km/s (blue), 0km/s < *V*_DF_<150km/ s (magenta), and *V*_DF_>150km/s (red). The number of events in each velocity bin is given in Table 1 and in the legend of Figure 3.

**Table 1:** Number of Events in Each Velocity Bin, the Temporal Scale of the DFs With 95% Confidence Bounds Obtained From the Linear Regression, and the Mean DF Thickness With Standard Deviation

	DF Velocity	Number of Events	Temporal Scale (s)	DF Size (km)
	*V*_DF_>150 km/s	8(35 %)	33 ± 30	9600 ± 8000
Cluster	0km/s < *V*_DF_<150km/s	9(39 %)	45 ± 27	3700 ± 2200
	−150km/s < *V*_DF_<0km/s	6(26 %)	42 ± 32	1900 ± 1000
	*V*_DF_<−150km/s	–	–	–
	*V*_DF_>150km/s	13(57 %)	11 ± 7	4400 ± 3200
MMS	0km/s < *V*_DF_<150km/s	5(21 %)	15 ± 8	1200 ± 700
	−150km/s < *V*_DF_<0km/s	3(13 %)	17 ± 10	1100 ± 900
	*V*_DF_<−150km/s	2(9 %)	10	2700 ± 400

The first major result is that at MMS about ∼57 % of the DFs move faster than 150km/s, while at Cluster only ∼35 % fall into this group, although the background *B*_*z*_, −3min to −2min, before the DF passage, is generally about ∼3 nT ± 1 nT higher at MMS (see Figure 3a). Furthermore, Cluster observes no fast tailward moving DFs (*V*_DF_<−150km/s). Note that the negative DF velocities correspond to tailward moving DFs (blue and black lines). The superposed epoch analysis of *B*_*z*_ also reveals that for Cluster the time between *B*_*z*,min_ and *B*_*z*,max_ of the earthward propagating DFs (magenta and red lines) decreases with enhanced DF velocity. For MMS, however, the fast and moderately earthward propagating DFs show a similar temporal behavior. Moreover, MMS shows a deeper decrease before the DF and a larger overshoot after the DF compared to Cluster.

As the second major result, we find that the *B*_*z*_ of the fast and moderately earthward moving DFs start to differ significantly ∼60 s before the DF passage (see Figure 3b). At both Cluster and MMS, the mean *B*_*z*_ before the fast DFs is higher than before the slowly propagating DFs.

Furthermore, we find that for the events of moderate velocity, *E*_*y*,T89_ is smaller, which suggest only a small flux transport rate in *X*_T89_ direction. We also find a strong negative *E*_*y*,T89_ for the fast tailward propagating MMS events, which is, however, only about half as large as *E*_*y*,T89_ for the earthward propagating events. This indicates that less flux is transported tailward.

In addition, MMS observes slightly higher elevation angles before crossings of earthward moving DFs than Cluster, indicating a slightly more dipolarized field configuration before the DF passage. The elevation angles of the fast‐moving DFs, particularly before the DF crossings are higher than those of the slower‐moving DFs. Moreover, Cluster sees a larger change in magnetic elevation angles across the DFs, corresponding with a larger change from a more tail‐like to a more dipolar‐like field configuration. At MMS, however, this behavior is less pronounced. Interestingly, tailward moving DFs at MMS show significantly higher elevation angles before the DF than Cluster.

We also examine the relationship between the DF velocity and thickness. The slope of linear fits to *V*_DF_ versus DF_size_ yields the temporal scale of the DFs. They are summarized in Table 1 and reveal that (1) fast‐propagating DFs have smaller temporal scales but larger DF thicknesses than slower‐propagating DFs and (2) DF thicknesses and temporal scales are generally larger at Cluster than at MMS.

## 5 Discussion

At MMS and Cluster about three quarters of the observed DFs propagate earthward and about one quarter tailward. This is in good agreement with earlier results from *Schmid et al.* [2011], who used Cluster observations between 2001 and 2007 and found that more than two thirds of the studied events propagate earthward.

Typically, flow braking occurs in regions of higher background *B*_*z*_. To evaluate the background conditions reliably, the average *B*_*z*_ and elevation angles during the interval 3–2 min before the DFs are estimated. Indeed, MMS observes slightly larger background *B*_*z*_ and elevation angles (by ∼3 nT ± 1 nT and ∼8° ± 4°) than Cluster, indicating that MMS was in a more dipolar background magnetic field. We might expect that the fast‐moving DFs at Cluster evolve into moderate‐moving DFs at MMS due to the flow braking. Interestingly, however, at MMS ∼57 % of the studied DFs propagate faster than 150km/s, while at Cluster only ∼35 % of the DFs fall in this group. This contradicts the idea that a DF motion becomes slower when propagating earthward if these numbers should reflect a single flow evolution. A possible explanation for this unexpected behavior might be that MMS and Cluster observed DFs at different conditions:

1. The tail season for MMS is between March and July, while for Cluster it is between July and October. Thus, the plasma sheet tilt is different, which may affect the location of the flow‐braking region.
2. Due to the small sample size, there might be a solar wind and/or solar cycle dependence in the data set. *Nagai et al.* [2005] showed that the solar wind *V*_*x*_ *B*_south_ controls the radial distance of the reconnection site in the magnetotail: magnetic reconnection takes place closer to the Earth when *V*_*x*_ *B*_south_ is higher. Indeed, using the mean of the 1 min OMNI data over 15 min before the DF events, we find on average a higher *V*_*x*_ *B*_south_ value at MMS (1.1 mV/m) than at Cluster (0.6 mV/m).
3. Since MMS might be located closer to the flow‐braking region, only DFBs with an entropy much lower than the surrounding plasma can be observed. According to the “plasma bubble” theory [see *Wolf et al.*, 2009], those DFB penetrate deeper into the near‐Earth plasma sheet with higher velocities. Indeed, *Shiokawa et al.* [1997] showed that although the occurrence rate of the high‐speed flows substantially decreases when the satellite comes closer to the Earth until 10 *R*_*E*_, but then slightly increases inside of 10 *R*_*E*_ (see their Figure 1a).
4. MMS may observe only a selection of DFs, those with an enhanced magnetic tension force or a reduced pressure gradient force. As shown by *Shiokawa et al.* [1997], the earthward flow can be easily braked within a few *R*_*E*_ under the typical tailward pressure gradient force of 1.2 × 10^−17^ Pa/m. Thus, either reduced tailward pressure gradient force or higher acceleration by enhanced earthward magnetic tension force is necessary to transport DFs from the reconnection region outside 20 *R*_*E*_ to inside 12 *R*_*E*_. The DF velocity at the flow‐braking region seems therefore more variable than stopping at one distance.

An important implication of the high‐velocity DFs at MMS is that these events transport a high amount of magnetic flux, as evidenced by the high *E*_*y*,T89_ (see Figure 3c), although located in a more dipolar field region. This fact indicates that a strong magnetic flux transport can take place even in the inner magnetosphere. *Nakamura et al.* [2009] showed that the flux transport rate, obtained from the timing velocity, ion flow velocity, and electric field measurements are quite consistent. Here *E*_*y*,T89_ is determined from *V*_DF_ and not from the plasma flow velocity or direct electric field measurements. Hence, it only reflects the flux transport rate properly, if the plasma flow velocity corresponds to the DF velocity.

Furthermore, larger DF velocities actually correspond to higher *B*_*z*_ values just before the DFs (see Figure 3b). The interesting point is that both spacecraft missions observe this behavior, although they are located in different regions (more/less dipolar magnetic field). This suggests that the increased ambient *B*_*z*_, from −60 s to −10 s ahead of the DF, exhibit rather local than global characteristics: the ambient *B*_*z*_ represents a local property of the magnetic field before the DF. This behavior has also been reported by *Nakamura et al.* [2009] who studied the flux transport in the tail and investigated pulses of DFs. We interpret that the higher ambient *B*_*z*_ originates from a magnetic flux pileup in the plasma, caused by the already increased plasma velocity in front of the DF. The increased plasma flow ahead of the DF is a result of the remote sensing of the approaching DF by the plasma, similar to a snowplow accumulating and pushing the snow ahead of it. In a superposed epoch analysis *Runov et al.* [2009] showed that the plasma velocity increases gradually, starting ∼40 s before the DF. This is in good agreement with our results, since the mean *B*_*z*_ starts to significantly differ ∼60 s ahead of the front.

There is also a significant number of tailward moving DFs observed from both, Cluster and MMS. Since it is unreasonable to assume reconnection so close to Earth, the tailward propagating events are the result of a DF rebound (bouncing) at the magnetic dipole‐dominated near‐Earth plasma sheet: The fast‐moving DFs get first compressed at the dipole‐dominated region and are then reflected tailward [e.g., *Panov et al.*, 2010; *Birn et al.*, 2011]. Indeed we observe compressed DFs with smaller temporal scales and spatial thicknesses at MMS than at Cluster. As the DFs move tailward, the magnetic tension force slows them down. In agreement with this picture, there are no fast tailward moving DFs at Cluster. Only MMS observes fast tailward propagating DFs, with high elevation angles before the DFs. We interpret the high elevation angles as the remnants of previously earthward propagating DFs. Thus, we suggest that the fast tailward moving DFs are recorded directly after the rebound of the fast earthward moving DFs.

The results obtained in this study are subject to a number of assumptions: (1) the DFs have a semicircular geometry, which is stable during the DF passage over all spacecraft; (2) the scales of the DFs are much larger than the probes separations; and (3) the DFs are propelled by the magnetic tension force and thus propagate along the magnetic field line direction in the lobes (above and below each observation location), projected onto the *X* *Y* GSM plane. In general the DF propagation direction is different from the DF crossing normal direction. Hence, the estimated timing velocity is only a projection (underestimation) of the actual DF velocity. Thus, we deproject this velocity onto the assumed DF propagation direction. To keep deprojection errors low, we require that the S/C cross the DFs at a maximal cone angle of 45° around this propagation direction. The time lags between the spacecraft are clearly larger than the data resolution and are thus a rather small uncertainty factor in the DF velocity determination. However, our findings can only be interpreted in the context of the aforementioned assumptions. In reality, the DF propagation and structure might be much more complicated, as their geometry might not be stable and they might expand as they propagate.

## 6 Summary and Conclusion

Assuming the DF to be a stable, semicircular structure, propagating along the magnetic tension force, the major results obtained in this study are as follows:

1. A larger fraction of the DFs move faster closer toward Earth than farther down the tail. This is contrary to the expectation that the DFs and associated DFBs should be braking in a more dipolar field where the flux tube entropy of the DFBs equals the entropy of the surrounding plasma. Here we discuss different alternatives for this behavior. First, a temporal selection of the DFs due to different solar wind conditions and/or plasma sheet tilting angles could have taken place. It is also possible that we only observe a selection of DFs closer to Earth, those with higher velocities in the first place. Clearly, a much larger data set of DFs is necessary to determine which mechanism is responsible for the unexpected behavior of the DFs close to Earth.
2. Larger DF velocities actually correspond to higher *B*_*z*_ values directly ahead of the DFs. This behavior is observed by both Cluster and MMS, although they are located in different regions in the tail (more/less dipolar magnetic field). We interpret the higher *B*_*z*_ to a local snow plow‐like phenomenon resulting from a higher DF velocity and thus a higher magnetic flux pileup ahead of the DF.

## Supporting information



Supporting Information S1

Data Set S1
