# Supplementary material for: A comparative study of dipolarization fronts at MMS and Cluster
Source: Geophys Res Lett. 2016 Jun 25;43(12):6012–9. doi: 10.1002/2016GL069520 (PMC4949994; doi:10.1002/2016GL069520)
Supplement: Supplementary file 1 — Supporting Information S1 [file GRL-43-6012-s001.pdf]

% README.pdf

The file DF_events.txt in the supplementary material contains a list of the studied DFs in the following format:

First list (DFs observed by MMS): DD-MMM-YYYY HH:MM

Second list (DFs observed by Cluster): DD-MMM-YYYY HH:MM
